# Dental Occlusion in a Split Amazon Indigenous Population: Genetics Prevails over Environment

**DOI:** 10.1371/journal.pone.0028387

**Published:** 2011-12-22

**Authors:** David Normando, Jorge Faber, João Farias Guerreiro, Cátia Cardoso Abdo Quintão

**Affiliations:** 1 Department of Orthodontics, Dental School, Federal University of Pará, Belem, Brasil; 2 Department of Orthodontics, Faculty of Health Science, University of Brasilia, Brasilia, Brasil; 3 Laboratory of Human and Medical Genetics, Center of Biological Sciences, Federal University of Pará, Belem, Brasil; 4 Department of Orthodontics, Dental School, State University of Rio de Janeiro, Rio de Janeiro, Brasil; University of Utah, United States of America

## Abstract

**Background:**

Studies examining human and nonhuman primates have supported the hypothesis that the recent increase in the occurrence of misalignment of teeth and/or incorrect relation of dental arches, named dental malocclusion, is mainly attributed to the availability of a more processed diet and the reduced need for powerful masticatory action. For the first time on live human populations, genetic and tooth wear influences on occlusal variation were examined in a split indigenous population. The Arara-Iriri people are descendants of a single couple expelled from a larger village. In the resultant village, expansion occurred through the mating of close relatives, resulting in marked genetic cohesion with substantial genetic differences.

**Methodology/Principal Findings:**

Dental malocclusion, tooth wear and inbreeding coefficient were evaluated. The sample examined was composed of 176 individuals from both villages. Prevalence Ratio and descriptive differences in the outcomes frequency for each developmental stage of the dentition were considered. Statistical differences between the villages were examined using the chi-square test or Fisher's exact statistic. Tooth wear and the inbreeding coefficient (*F*) between the villages was tested with Mann-Whitney statistics. All the statistics were performed using two-tailed distribution at p≤0.05. The coefficient inbreeding *(F)* confirmed the frequent incestuous unions among the Arara-Iriri indigenous group. Despite the tooth wear similarities, we found a striking difference in occlusal patterns between the two Arara villages. In the original village, dental malocclusion was present in about one third of the population; whilst in the resultant village, the occurrence was almost doubled. Furthermore, the morphological characteristics of malocclusion were strongly different between the groups.

**Conclusions/Significance:**

Our findings downplay the widespread influence of tooth wear, a direct evidence of what an individual ate in the past, on occlusal variation of living human populations. They also suggest that genetics plays the most important role on dental malocclusion etiology.

## Introduction

Human dentition may adapt functionally to diet and feeding behavior. Bones continue to remodel throughout an individual's life, but tooth enamel does not regenerate once its formation is complete. For these reasons, dental morphology has been studied as an important indicator of both functional adaptations and phylogenetic evolution [1]–[5].

Dental malocclusion is a misalignment of the human dentition, either due to overcrowding within each dental arch or an abnormal relationship between both dental arches. Despite the millions of people around the world undergoing orthodontic treatment, the etiology of dental malocclusion is still not clear [6]. Although little is known about its origin in human populations, dental malocclusion has been often referred to as a “disease of civilization” [7], [8]. Studies examining human [7]–[13] and nonhuman primates [14]–[17] have indicated that the increase in the occurrence of dental malocclusion can be attributed to the availability of a more processed diet and a reduced need for powerful masticatory action. Although there is also some weak evidence suggesting genetic influences on the occurrence of malocclusion in *Homo sapiens* [18]–[20], these studies have relied on the extrapolation of data from the remains of ancient populations for which we cannot determine the cause of death.

**Figure 1:**
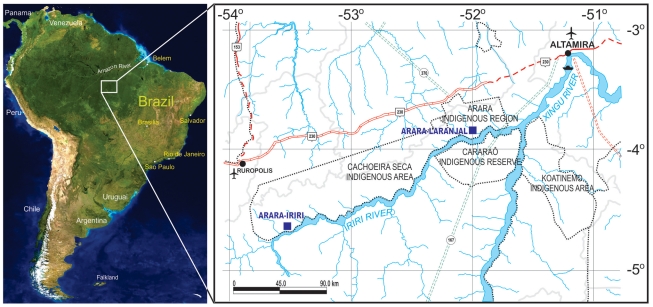
Map of South America. The magnified box highlights the Xingu river region and its tributary, the Iriri River, where the Arara-Laranjal and Arara-Iriri villages are located. The city of Altamira (top right) is the nearest town and is located 120 km and 320 km upstream of the Laranjal and Iriri villages, respectively.

The Arara-Iriri indigenous group lives by the Xingu River in the Amazon region and was first contacted in 1987. A previous anthropological study [21] reports that the people who constitute this village are descendants of a single couple who were expelled about 90 years ago from a larger village. The larger village, named Laranjal, was first contacted in 1983 and is located 200 kilometers away [Fig. 1]. The presence of a single Y chromosome, one mtDNA haplotype, and only one to four alleles at all autosomal loci studied confirmed an extreme case of lineal fission involving related individuals from an ancestral village [22].

Increased tooth wear is considered a phenotypic signal of an adaptive shift to exploit tougher and more abrasive food resources. Tooth wear has been used to describe hominid evolution [1], [2], [23], [24] because it provides direct evidence of what an individual ate in the past [23], [24]. The eating habits of the indigenous tribes inhabiting the Xingu region are predominantly traditional and usually based on cassava, nuts, fish, meat of wild animals, sweet potatoes, yams and wild fruits [25]. All children are breastfed until the birth of the next child, which usually occurs after 1.5–2 years.

Although small founder populations seem to be frequent events in the formation of new tribes among the Amazon Amerindians, the fission process and its consequences on the differentiation of ethnic groups have seldom been recorded. In addition, the influence of founder effects on dentition has never been reported. Given the accessibility of historical [21] and genetic records [22] for these two human populations, the examination of the Arara villages provides a unique opportunity to improve our knowledge about the etiology of dental malocclusion.

## Results

While the Arara-Laranjal village was expanded by nonconsanguineous or rare incestuous relations, the initial expansion of the Arara-Iriri group occurred through the mating of closely related people, including parents and their offspring and sibling with their siblings, and later by marriages between relatives somewhat more distant, such as uncle-niece, aunt-nephew and first cousins [22]. The median degree of inbreeding as measured by the inbreeding coefficient (*F*), for the Arara-Iriri village was 0.25 (IQR = 0.25) and differed significantly (p<0.0001) from the Arara-Laranjal population, whose median is 0 (IQR = 0). This finding confirms the frequent incestuous unions among the Arara-Iriri indigenous group.

**Figure 2:**
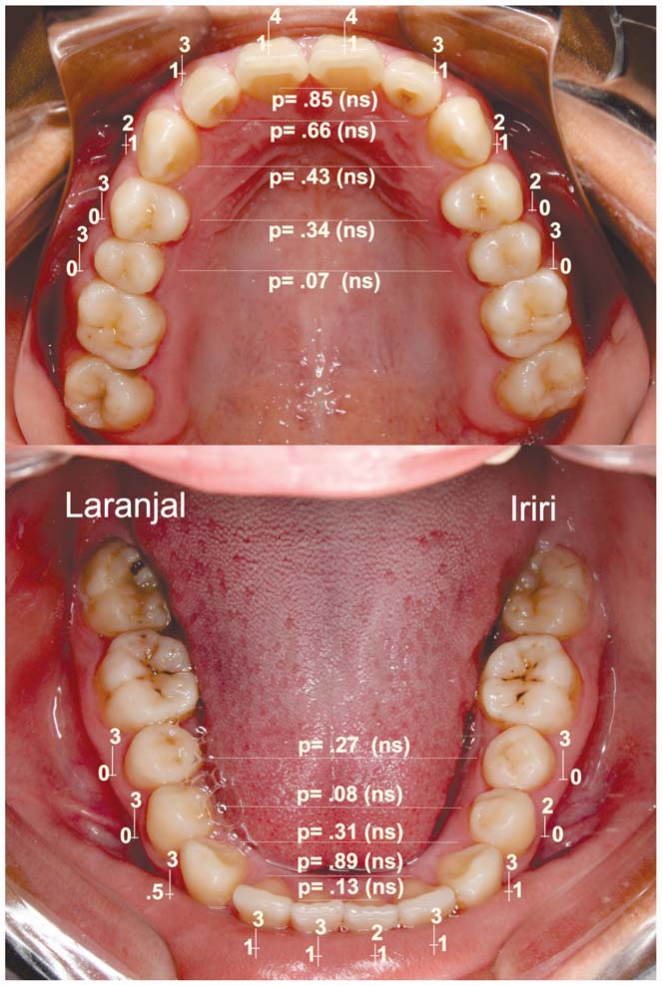
Descriptive statistics for tooth wear in the upper and lower jaw. Median (−), minimum and maximum values for the Arara-Laranjal (left side, n = 58) and Arara-Iriri (right side, n = 23) populations. P values were obtained using a Mann-Whitney test.

Regarding tooth wear evaluation we observed a very similar pattern for both villages (Figure 2), confirming the similarity of the eating habits among the Xingu indigenous groups [25].

**Figure 3:**
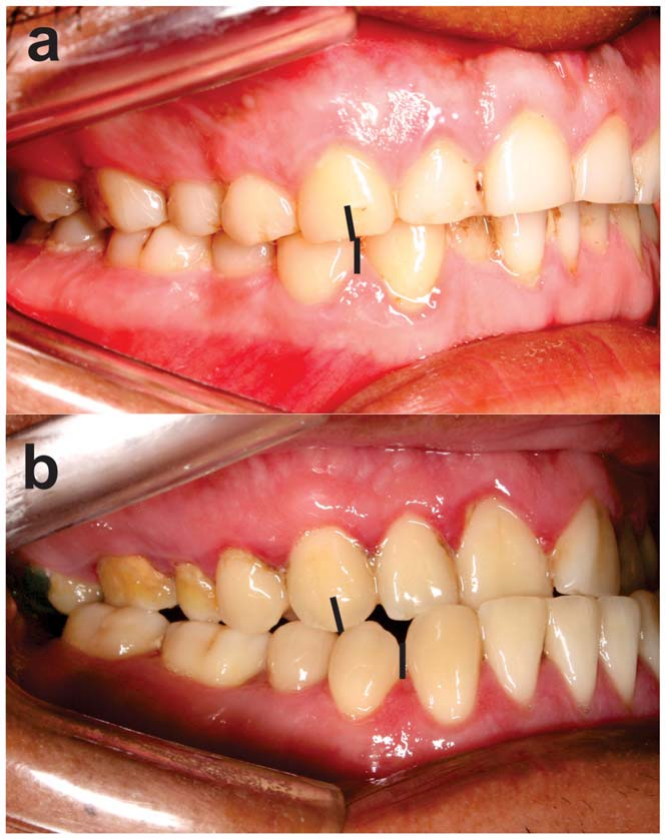
Dental occlusion in a split Amazon indigenous population. **A**) Normal occlusion as observed in a male individual from the Arara-Laranjal village. The lines indicate the upper canine tip occluding between the lower canine and lower 1^st^ premolar. **B**) Class III malocclusion associated with anterior and posterior crossbite in a male from the Arara-Iriri village. The lines indicate a misaligned upper canine tip occluding posterior to the lower canine and lower 1^st^ premolar.

In the original village (Laranjal), only one third of the population showed dental malocclusion (33.8%). By contrast, in the resultant village, the rate of malocclusion was nearly doubled (63%; prevalence ratio (PR) = 1.86, p = 0.0005). Normal occlusion was the most common morphological pattern in the original Arara village (Table 1 and Figure 3A), but for the Arara-Iriri indigenous people, the common pattern was a Class III malocclusion in which the lower dentition is more prominent than the upper dentition (32.6%, PR = 6.1, p<0.0001) (Table 1 and Fig. 3B). Class II malocclusion, diagnosed when the upper dentition is more prominent than the lower dentition, was also two times higher in the resultant village. Anterior open bite (PR = 2.64, p = 0.003), anterior crossbite (PR = 2.83, p<0.001), overjet (PR = 3.39, p = 0.03) and posterior crossbite (PR = 4.71, p = 0.02) were also more common in the Arara-Iriri village. Although no subject presented dental crowding or overbite in the resultant village, overbite was evident in 5 individuals (3.9%), and dental crowding was observed in 20 individuals (20.8%, p = 0.005) in the original village. These data confirm a marked divergence between the villages for dental malocclusion prevalence.

**Table 1:** Dentition features in the Arara-Iriri and Arara-Laranjal groups for deciduous, mixed and permanent dentition.

	Iriri Village				Laranjal Village				
	Deciduous n = 8 (17.4%)	Mixed n = 15 (32.6%)	Permanent n = 23 (50%)	TOTAL n = 46 (100%)	Deciduous n = 34 (26.2%)	Mixed n = 38 (29.2%)	Permanent n = 58 (44.6%)	TOTAL n = 130 (100%)	PR p-value
***Normal X Malocclusion***								1.86***	
Normal	5 (62.5%)	4 (26.7%)	8 (34.8%)	17 (37.0%)	28 (82.4%)	21 (55.3%)	37 (63.8%)	86 (66.2%)	
Malocclusion	3 (37.5%)	11 (73.3%)	15 (65.2%)	29 (63.0%)	6 (17.6%)	17 (44.7%)	21 (36.2%)	44 (33.8%)	
***Malocclusion Classif.***									
Class I	1 (12.5%)	1 (6.7%)	2 (8.7%)	4 (8.7%)	3 (8.8%)	9 (23.7%)	11 (19.0%)	23 (17.7%)	0.58 ns
Class II	1 (12.5%)	4 (24.7%)	5 (21.7%)	10 (21.7%)	2 (5.9%)	7 (18.4%)	5 (8.6%)	14 (10.8%)	2.0*
Class III	1 (12.5%)	6 (40%)	8 (34.8%)	15 (32.6%)	1 (2.9%)	1 (2.6%)	5 (8.6%)	7 (5.4%)	6.1***
***Malocclusion Type***									
Overbite	0 (0.0%)	0 (0.0%)	0 (0.0%)	0 (0.0%)	1 (2.9%)	4 (10.5%)	0 (0.0%)	5 (3.9%)	∞ ns
Open bite	1 (12.5%)	4 (26.7%)	9 (39.1%)	14 (23.9%)	2 (5.9%)	5 (13.2%)	8 (13.8%)	15 (11.5%)	2.64**
Overjet	1 (12.5%)	2 (13.3%)	3 (15.8%)	6 (14.3%)	1 (2.9%)	2 (5.2%)	2 (3.4%)	5 (3.9%)	3.39*
Anterior Crossbite	1 (12.5%)	7 (46.7%)	8 (34.8%)	16 (30.4%)	2 (5.9%)	3 (7.9%)	11 (19.0%)	16 (12.3%)	2.83***
Posterior Crossbite	1 (12.5%)	3 (20.0%)	1 (5.3%)	5 (11.9%)	1 (2.9%)	1 (2.6%)	1 (1.7%)	3 (2.3%)	4.71*
Midline deviation	NA	0 (0.0%)	6 (26.1%)	6 (15.8%)	NA	4 (10.5%)	9 (15.5%)	13 (13.6%)	1.17 ns
Crowding	NA	0 (0.0%)	0 (0.0%)	0 (0.0%)	NA	9 (23.7%)	11 (19.0%)	20 (20.8%)	∞ **
Spacing	NA	1 (6.7%)	0 (0.0%)	1 (2.6%)	NA	0 (0.0%)	0 (0.0%)	0 (0.0%)	∞ ns

PR = Prevalence Ratio.

∞ PR Not computed (zero)/p-value for NA = Not available (ns) = not significant;

* P<.05;

** P<.01;

*** P<.001.

## Discussion

Scientifically, the role of environment and genetics on dental malocclusion has been discussed under three models of study designs: the analysis of skull remains of ancient populations [7]–[13], experiments with animal models [14]–[17] and research on human twins [26]–[30]. Most of these investigations have advocated that the increase in the occurrence of dental malocclusion must be attributed to environment changes during human evolution [7]–[17], [27]–[28].

Some studies examining twins have suggested dental malocclusion as a primary consequence of environmental changes [27], [28]. Recently, several criticisms have been attributed to this research model [26]. A major issue of concern in many previous studies of twins has been the accuracy of zygosity determination by comparisons of physical appearance. More recently, the use of highly polymorphic regions of DNA has proved to be more accurate and reliable [30].

We examined the genetic and dietary influences through tooth wear on occlusal variation between two split indigenous villages. Our findings revealed that two indigenous populations belonging to the same ethnicity showed marked differences regarding dental malocclusion prevalence (Table 1). From deciduos to permanent dentition, normal occlusion was the most common morphological pattern in the original Arara village (Table 1 and Figure 3A), but for the Arara-Iriri indigenous people, the common pattern was the presence of dental malocclusion. These findings should not be attributed to dietary consistence, as reports [25] have pointed out similar feeding habits for both villages; these findings were corroborated by similar patterns of tooth wear found in the present investigation (Figure 2). Findings strongly suggest that the marked difference in dental occlusion is related to the genetic distance and molecular variance between the two Arara villages, as previously reported [22].

The Amerindian population exhibits genetic diversity as high as that of any other human population; the large variation between tribes compensates for the low variation within tribes, a feature attributed to the genetic drift acting on small, isolated populations [22]. While a genetic investigation confirmed that the Arara-Iriri village was founded by a single couple who came from the ancestral village [22], the molecular variance between these two contemporary tribes was greater than that observed between them and other Amazonian tribes [22], [31]. The fission process, which was influenced by a marked genetic cohesion and a dramatic founder effect, has produced remarkable genetic differences between the villages. Therefore, despite their common origin, the populations that constitute these villages have different genotypes.

In light of similar tooth wear, an outcome with a pronounced influence of the environment, the striking difference in the dental malocclusion between the Arara indicates that genetics substantially impacts the morphology of the facial bones and dentition. Moreover, our results suggest that a founder effect and genetic inbreeding have exacerbated alterations in dental occlusion in the Arara-Iriri village population. These findings lend support to the notion that variation in continuous quantitative traits is usually polygenic [32], [33] and that we should expect a highly polygenic basis for complex traits such as human craniofacial and dentition morphology and development [33]. Moreover, our findings downplay the widespread influence of tooth wear on dental malocclusion in current human populations [7]–[17].

## Materials and Methods

This study was approved by the National Ethical Committee for Health Sciences of the Brazil registered under number 25000.066559/2010-11. Written Informed consent was obtained prior to data collection.

The total population was composed of 239 individuals living in the Arara-Laranjal village and 80 individuals living in the Arara-Iriri village. The number of subjects not present due to hunting or fishing expeditions during the study period was 11 (4.6%) from the Laranjal tribe and 6 (7.5%) from the Iriri tribe. All individuals present were examined, but only those subjects aged between 2 and 22 years old have been included in this analysis. Therefore, the sample was composed of 130 individuals from the Laranjal tribe and 46 from the Iriri village (Table 2). Tooth wear was examined only for individuals with permanent dentition (n = 81).

**Table 2:** Sample size (n) and mean age (years) in the Arara-Laranjal and Arara-Iriri villages.

	Arara-Iriri (n = 46)				Arara-Laranjal (n = 130)			
	Male		Female		Male		Female	
*Dentition*	n (%)	Age- yrs	n (%)	Age- yrs	n (%)	Age- yrs	n (%)	Age- yrs
Deciduous	2 (20%)	3.2	6 (80%)	3.3	20 (58.8%)	4.3	14 (42.2%)	4.2
Mixed	5 (33.3%)	8.6	10 (66.7%)	7.6	24 (63.2%)	8.3	14 (36.8%)	8.7
Permanent	9 (39.2%)	16.8	14 (60.1%)	15.6	26 (44.8%)	16.6	32 (55.2%)	15.3
**TOTAL**	16 (34.8%)	12.5	30 (65.2%)	10.5	70 (53.4%)	10.2	60 (46.2%)	11.2

### Clinical Examination

The dental examination was performed by the same examiner, an orthodontist with expertise in public health and 20 years of experience. The examination was performed using natural daylight and a flashlight, with an assistant recording the observations. The tooth wear scores [19] and the dental malocclusion [34], [35] were categorized as in previous studies.

Several morphological characteristics were examined during the clinical examination:

- stage of dentition as deciduous, mixed or permanent;
- early loss of deciduous and/or permanent teeth;
- dental anomalies;
- right and left molar sagittal relationship in the permanent dentition classified as Class I, Class II or III;
- right and left canine sagittal relationship, in the deciduous and mixed dentitions scored as Class I, Class II or III;
- incisal relationship for overjet and/or overbite, scored as normal, increased, anterior crossbite or open bite;
- transverse arch relationship defined as normal or posterior crossbite;
- upper and lower midline noted as normal or deviated;
- alignment of the upper and lower arches as normal, spacing or crowded.

The subjects were classified as having a malocclusion when one or more of the following occlusal characteristics were present, according to previously described methods [34], [35]:

- Class II or Class III molar sagittal relationship (permanent dentition) or canine relationship (deciduous and mixed dentition);
- posterior or anterior crossbite;
- overjet >3 mm;
- overbite <1 mm or >4 mm;
- midline deviation >2 mm;
- crowding or spacing larger than 3 mm in the permanent dentition;
- early deciduous or permanent tooth loss.

### Tooth wear examination

Tooth wear was examined in the permanent dentition through a slight modification of the classification system previously described [19]. The occlusal surfaces of the second and first premolars, canines and lateral and central incisors in both arches were examined with the aid of a flashlight. The following scores were recorded for each tooth: 0 = no wear; stage 1 = wear of the enamel only; stage 2 = wear of the dentin, where the occlusal surface had more enamel than dentin; stage 3 = wear of the dentin where the occlusal surface presents more dentin than enamel; or stage 4 = advanced wear stage, near the pulp and beyond.

### Statistical Analysis

Statistical differences between the two villages were examined using the chi-squared test or Fisher's exact statistic according to the expected values. Because one of the tribes had a relatively small population, the gender and developmental stage groups were pooled for the statistical comparison of frequency data. Furthermore, the prevalence ratio (PR) and descriptive differences in the outcome frequency for each dentition stage were also considered. The statistical differences between the villages for the dental wear of each tooth and the inbreeding coefficient (*F*) were tested using a Mann-Whitney test. All statistics were performed using two-tailed distribution at p≤0.05 through the BioEstat statistical software (version 5.0, Mamirauá Maintainable Development Institute, Belém, Pará, Brazil).

Duplicate clinical examinations were carried out by the same examiner on a randomly selected subsample of 60 individuals (34%). Due to the long distance to reach the villages (around 3–4 days travel from Altamira city by small motorboat), plaster dental casts and intraoral photographs were obtained. The test-retest was performed using Kappa statistics to compare the oral examination and records and demonstrated that the diagnostic exam was highly reproducible (k = 0.88; Table 3).

**Table 3:** Kappa coefficient for diagnostic method.

Occlusal trait	Kappa		p-value
Normal X Malocclusion	0.88	Exc	<0.00001
Right Cuspid Class	0.81	Exc	<0.00001
Left Cuspid Class	0.94	Exc	<0.00001
Overbite/Anterior Open Bite	0.94	Exc	<0.00001
Overjet/Anterior crossbite	0.97	Exc	<0.00001
Posterior crossbite	0.66	Good	<0.00001
Upper midline deviation	0.97	Exc	<0.00001
Lower midline deviation	0.95	Exc	<0.00001
Upper Crowding/Spacing	0.90	Exc	<0.00001
Lower Crowding/Spacing	0.88	Exc	<0.00001
Tooth loss	1.00	Exc	<0.00001

Intraclass correlation was used to test the reliability of the tooth wear evaluation. The mean scores obtained during the clinical evaluation were compared to those obtained from the occlusal photographs of 20 subjects. The tooth wear measurements also showed excellent reproducibility (r = 0.78–0.94, p<0.0001). However, among the 20 pairs of ratings, the values obtained when examining tooth wear via occlusal photographs were slightly higher in 18 of the evaluations. These findings indicate that this method tends to slightly overestimate the level of tooth wear when compared to direct clinical examination.
